# Machine Learning Data Augmentation as a Tool to Enhance Quantitative Composition–Activity Relationships of Complex Mixtures. A New Application to Dissect the Role of Main Chemical Components in Bioactive Essential Oils

**DOI:** 10.3390/molecules26206279

**Published:** 2021-10-17

**Authors:** Alessio Ragno, Anna Baldisserotto, Lorenzo Antonini, Manuela Sabatino, Filippo Sapienza, Erika Baldini, Raissa Buzzi, Silvia Vertuani, Stefano Manfredini

**Affiliations:** 1 Department of Computer, Control, and Management Engineering “Antonio Ruberti”, Sapienza University, 00185 Rome, Italy; alessio.ragno@uniroma1.it; 2 Department of Life Sciences and Biotechnology, University of Ferrara, 44100 Ferrara, Italy; anna.baldisserotto@unife.it (A.B.); bldrke@unife.it (E.B.); bzzrss@unife.it (R.B.); 3 Department of Drug Chemistry and Technology, Sapienza University, 00185 Rome, Italy; lorenzo.antonini@uniroma1.it (L.A.); manuela.sabatino@uniroma1.it (M.S.); filosapi@gmail.com (F.S.); 4 Master Course in Cosmetic Sciences, Department of Life Sciences and Biotechnology, University of Ferrara, 44100 Ferrara, Italy

**Keywords:** QCAR, machine learning, deep learning, essential oils, pharmaceutics, nutraceutics, cosmeceutics

## Abstract

Scientific investigation on essential oils composition and the related biological profile are continuously growing. Nevertheless, only a few studies have been performed on the relationships between chemical composition and biological data. Herein, the investigation of 61 assayed essential oils is reported focusing on their inhibition activity against *Microsporum* spp. including development of machine learning models with the aim of highlining the possible chemical components mainly related to the inhibitory potency. The application of machine learning and deep learning techniques for predictive and descriptive purposes have been applied successfully to many fields. Quantitative composition–activity relationships machine learning-based models were developed for the 61 essential oils tested as *Microsporum* spp. growth modulators. The models were built with in-house python scripts implementing data augmentation with the purpose of having a smoother flow between essential oils’ chemical compositions and biological data. High statistical coefficient values (Accuracy, Matthews correlation coefficient and F_1_ score) were obtained and model inspection permitted to detect possible specific roles related to some components of essential oils’ constituents. Robust machine learning models are far more useful tools to reveal data augmentation in comparison with raw data derived models. To the best of the authors knowledge this is the first report using data augmentation to highlight the role of complex mixture components, in particular a first application of these data will be for the development of ingredients in the dermo-cosmetic field investigating microbial species considering the urge for the use of natural preserving and acting antimicrobial agents.

## 1. Introduction

The origins of essential oils (EOs) dates back to at least a thousand years ago. They have been used and are still employed for medicinal, cosmetic, and nutritional purposes. EOs are concentrated oils derived from plants which are described as containing “the essence” of the plant. EOs can be derived from nearly any plant matter. EOs are mixtures of chemical components containing from a few to hundreds of compounds. In the latest 10–20 years there has been a growing interest in EOs as demonstrated by the thousands of articles published recently. More interestingly the global EOs demand was estimated at 247.08 kilotons in 2020 and it is expected to increase at a compound annual growth rate of 7.5% from 2020 to 2027 (https://www.grandviewresearch.com/industry-analysis/essential-oils-market accessed 22 July 2021). A few of the most commonly used EOs are tea tree oil, rosemary oil, and lavender oil. The EOs’ market value worldwide is expected to rise from around 17 billion U.S. dollars in 2017 to about 27 billion dollars by 2022 (https://www.statista.com/topics/5174/essential-oils/ 22 July 2021). Although thousands of scientific articles are published each year on EOs (scopus accessed 15 September 2021) only a few reported the application of machine learning (ML) algorithms with the purpose to dissect the chemical components which are mainly responsible for the associated biological profile to derive quantitative composition–activity relationships (QCAR) [1,2,3,4,5,6], while more commonly the use of ML to EOs was just confined in chromatography, to relate the chemical composition to the retention time of each compound [7,8,9].

The use of ML algorithms to develop predictive models has become popular in several scientific fields like drug design [10]. While it is often very easy to reach high performances with training data, it is very frequent to encounter problems in the testing phase. This is mostly due to experimental data, that are often noisy, poor and the derived models do not cover all the possible range of application, such as EOs’ chemical composition variability. To avoid the so-called “overfitting problem” caused by the great number of independent variables, in fields such as computer vision and signal processing, a common strategy is to generate new training records by means of perturbation of the original ones. Image data augmentation (DA) is generally performed in deep learning by flipping, rotating, and applying graphical filters to the training images [11].

A DA-based approach is reported in this study to analyze complex mixture using as case study a series of experimentally evaluated EOs. To the best of authors knowledge, this is the first report of DA application to EOs and in particular in the dermo-cosmetic field, where an increasing need for natural effective ingredients is occurring, especially in view of the guide-line of the “Green Deal”.

## 2. Overview of the Study

DA is of great importance for ML classification, particularly for biological data, which tends to be high dimensional and scarce. In this application we decided to apply DA to EOs to dynamically change their composition mixtures. This approach aims to tweak a very well-known Eos’ weak aspect, consisting in their difficulty to standardize the composition due to plants variation and extraction methods, often caused by the sensitivity of the composition variability due to the opening of containers. Nevertheless, minimal variation of chemical composition is known not to destroy the biological profile of the EOs. In this scenario the DA method includes such a variability with a reduced set of samples. This study proposes to apply DA to EOs by tweaking the composition percentages in order to perform statistical analysis on larger datasets, reducing overfitting and building reliable statistical models, this is not achievable with the raw dataset. Using the DA technique, it is possible to reshape unbalanced datasets by augmenting records proportionally to the class occurrences. Given the continuous interest in the dermo-cosmetic field, the proposed method has been applied to 61 EOs whose chemical compositions were already known [3,6] and here investigated for an anti-dermatophyte bioactivity as a model approach. The obtained ML models have been then used to dissect the chemical component’s role in the inhibition of the dermatophytes using perturbation-based techniques, impurity-based techniques, and coefficients analysis. The anti-dermatophyte activity of 61 EOs was investigated against *Microsporum* spp. (*Microsporum canis* (*M. canis*) and *Microsporum gypseum* (*M. gypseum.*)) through growth inhibition assay.

Dermatophytosis is an infection of keratinized tissues, nails, hair, and cornea extract, and is caused by several species belonging to the genera *Microsporum*, *Trichophyton,* and *Epidermophyton*, the only fungi able to invade and reside in keratinized tissues. They are transmitted by contact with hair and dandruff, both infected and containing fungal particles, coming from animals, the environment, or fomites. *M. gypseum* is a geophilic dermatophyte, as such it generally inhabits the soil, where it decomposes into keratinized debris. *M. canis*, on the other hand, being zoophilic, has adapted to animals and is only rarely found in the soil [12]. This pathology leads to health problems that are usually caused by topical drugs and/or dermo-cosmetic applications. Investigation on dermatophytes [13,14,15] reports the activity of a series of 61 commercial EOs (Table S1) on the growth inhibition of *Microsporum* spp.

## 3. Results

### 3.1. Antidermatophyte Activity of EOs

The percentage growth inhibition values of all the oils were tested at a concentration of 100 µg/mL (Table 1). Overall, the oils tested showed low growth inhibition on both dermatophytes. In fact, after treatment with different oils, increased fungal growth was observed for both *M. canis* and *M gypseum* (see + and ++ signs in Table 1) and more generally growth inhibition values well below 50%.

### 3.2. Application of Machine Learning Algorithms

#### 3.2.1. Model Definition

The model definition process has been performed by a thorough search using five different ML algorithms: logistic regression (LR), support vector machines (SVM), gradient boosting (GB), k-nearest neighbor (KNN), and random forest (RF) as implemented in the scikit-learn library [16]. For each experimental activity associated to the 61 EOs a grid search total of 4,050,000 models was performed to seek for the best ML algorithm (see Section 4.2 for details). The grid search was applied considering hyper-parameters variation, percentage of inhibition classification threshold, and the use of raw dataset or two data augmented versions of it (balanced and unbalanced). The models’ evaluation was performed by mean of the Matthew correlation coefficient (MCC) as it has been reported to be suited for unbalanced datasets [17].

##### *M. canis* Classification Modeling

**Figure 1:**
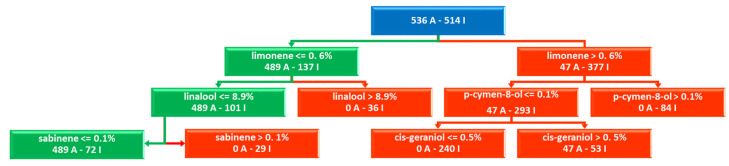
Top 3 levels of the RF first decision tree: most important chemical components from the DA dataset on *M. canis* bioactivities and EO virtualized chemical composition. The full tree is reported as Supplementary Figure S1. Green path indicates positive impact on *M. canis* inhibition, while red path, negative influence. The blue box indicates the starting DA dataset composition. In this tree limo-nene, linalool, and sabinene are indicated to be important but at low concentrations (lower than 0.6%, 8.9%, and 0.1%, respectively).

**Figure 2:**
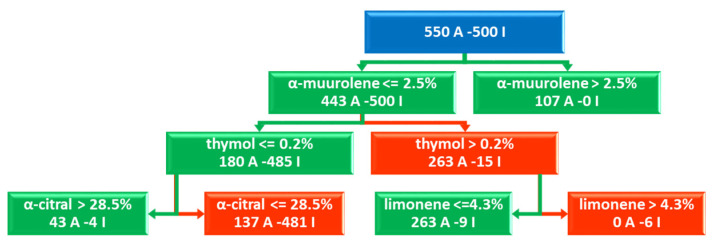
Top 3 levels of the RF second decision tree: most important chemical components from the DA dataset on *M. canis* bioactivities and EO-virtualized chemical composition. The full tree is reported as Supplementary Figure S2. Green path indicates positive impact on M. canis inhibition, while red path negative influence. The blue box indicates the starting DA dataset composition. In this tree, α-muurolene is indicated necessary for the inhibitory activity, thymol and limonene are important if at low concentrations (lower than 2.5% and 0.2%, respectively) and α-citral at high percentages (more than 28.5%).

The best model (ML1 in Table 2) was obtained with RF at a percentage of inhibition threshold set to 20% (11 actives and 50 inactives), using the balanced DA function and two random trees (estimators = 2, Table S2) (Figure 1 and Figure 2 and Supplementary Figures S1 and S2). The augmented dataset was composed of 1050 virtualized EOs mixtures with an active/inactive ratio of 536/514 for the first tree (Figure 1) and 550/500 for the second one (Figure 2). The model was characterized by optimal classification power and high cross-validated Accuracy_CV_, MCC_CV_, and F_1_ Score_CV_ classification coefficients values of 0.95, 0.984, and 0.97, respectively (Table 2 and Supplementary Table S2). For comparison purposes a model (ML2 in Table 2) using the raw dataset was also built using the same parameters leading to the following cross-validated coefficients: Accuracy_CV_ = 0.93; MCC_CV_ = 0.77; F_1_ Score_CV_ = 0.96.

##### *M. gypseum* Classification Modeling

As the percentage of inhibition potencies in the case of *M. gypseum* were higher than those for *M. canis* likely the grid-search optimization selected a cutoff value of 30% leading to an inactive/active ratio of 48/13. Differently from *M. canis*, the LR and SVM algorithms displayed the highest statistical coefficients (Table 3 and Supplementary Table S3). In particular LR showed slightly better accuracy and MCC coefficients than SVM, both displayed the same robustness in cross-validation runs. Contrarily to *M. canis* LR and SVM were not able to display any acceptable classification ability on the raw dataset of 61 essential oil as they returned MCC values lower than 0.5 (data not shown).

#### 3.2.2. Feature Importance Analysis

##### *M. canis* Classification Model

In the case of *M. canis* bioactivities, the best model (ML1) was obtained with the RF method composed of two decision trees, and inspection of the feature importance (chemical component importance) was performed following two methods indicated below:

*Impurity-based feature importance*: this sorts out the most relevant compounds which have higher information gain (entropy) measured through the Gini coefficient. This tool is very fast and easy to obtain, but on the other hand, it consists in a very biased approach when dealing with different kind of features. In fact, the impurity-based scores tend to inflate the relevance of continuous features or high cardinality categorical variables.
*Perturbation-based feature importance*: this measures the relevance by observing how random re-shuffling values influences the model performances. This approach turns out to be more reliable with different-shaped features but it is also more computationally expensive in comparison with the impurity-based method.

**Figure 3:**
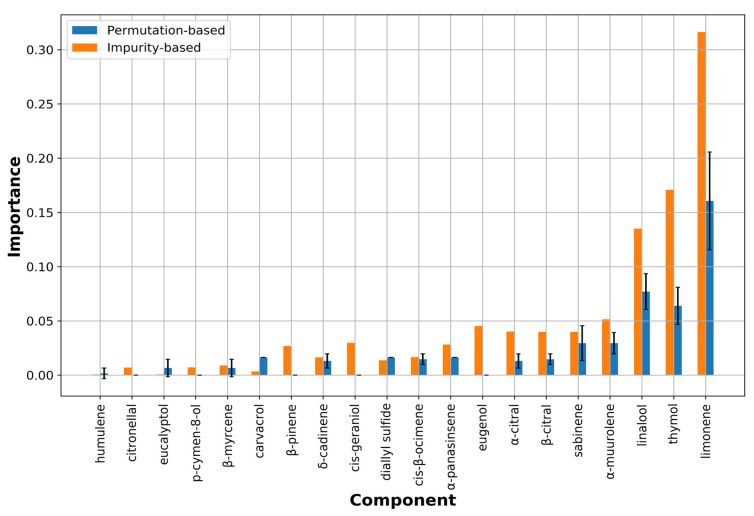
Feature importance (orange bars) for the 20 most important chemical components from the RF classification model applied to *M. canis* bioactivities. Perturbation-based importance (blue bars) is also reported using mean and standard deviation.

The analysis of the feature is partially quantitative being the values reported as absolute numbers giving no indication on which variable (chemical component) contributes positively or negatively to the activity (Figure 3).

Nevertheless, a sort of fully quantitative data could be obtained by analyzing the scikit-learn generated RF-learned trees from which it is possible to get indication on positive and negative influencing compounds on the anti-dermatophyte potency (Figure 2 and Figure 3 and Supplementary Figures S1 and S2).

Thus, analyzing impurity-based and perturbation-based methods with the RF trees, the most relevant components for the *M. canis* growth modulation, in decreasing/descending order, are, limonene, thymol, linalool, and α-muurolene that could exert all together a synergistic behavior, although for limonene and linalool a maximum of 0.6% and 8.9% are tolerated, respectively. On the other hand, p-cymen-8-ol and cis-geraniol presence might have some anti-synergistic impact on the *M. canis* growth and likely in some example could also contribute to the stimulation of dermatophyte growth (see discussion section). Other compounds could be highlighted as important but, the RF-associated trees are reported to be as relevant as more than twice than the others.

##### *M. gypseum* Model

**Figure 4:**
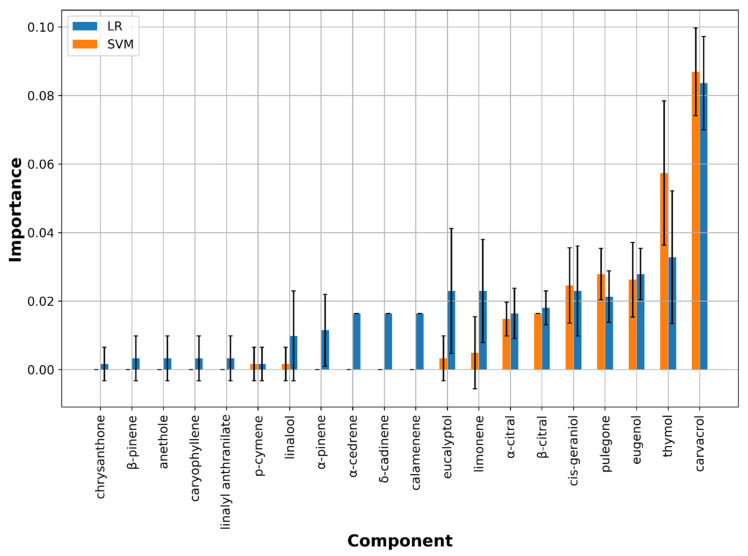
Perturbation-based feature importance for the LR (blue bars) and SVM (orange bars) ML-based models on *M. gypseum* reported using mean and standard deviation. Only the first 20 most important compounds are listed.

**Figure 5:**
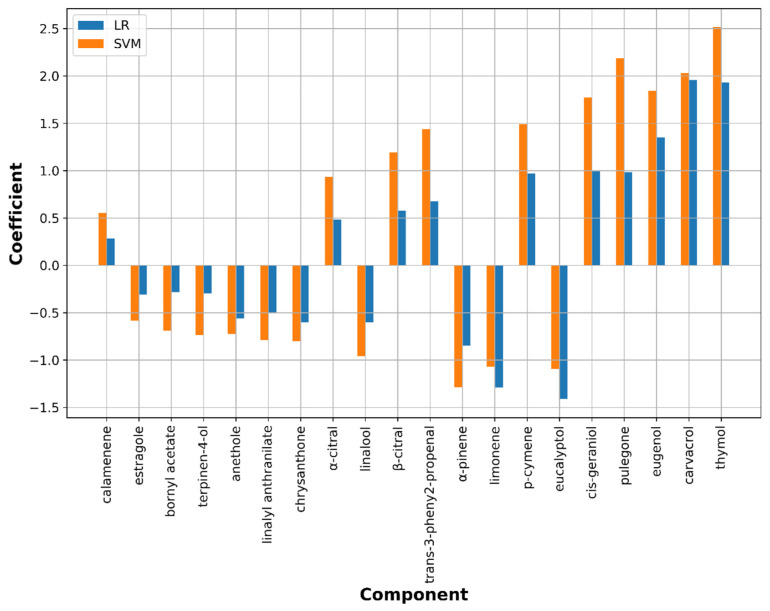
Linear coefficients for the LR and SVM ML-based models on *M. gypseum.* Only the first 20 most important compounds as in Figure 4 are displayed.

In the case of *M. gypseum*, two linear ML models (LR and SVM) resulted to be best classified as EOs and hence directly investigated through the chemical components importance. This was achieved by inspecting the linear coefficients from which it was possible to define the positive or negative impact of each component on dermatophyte growth inhibition/stimulation directly from the associated signs (positive or negative, respectively). Along with coefficient inspection, perturbation-based scores were calculated to compare the results from the two ML algorithms. Carvacrol, thymol, eugenol, pulegone, cis-geraniol, β-citral, α-citral, eucalyptol, and limonene were indicated as the most important compounds for the M. gypseum growth modulation (Figure 4). In particular, inspecting linear coefficient values carvacrol, thymol, eugenol, pulegone, cis-geraniol, β-citral, α-citral might have positive impact on the inhibition (Figure 5). On the contrary, compounds with medium importance and high coefficients (eucalyptol, limonene and α-pinene) were predicted to counteract inhibitory activity and likely could stimulate the dermatophyte growth.

## 4. Discussion

### 4.1. Effect of EOs on Dermatophytes growth

The samples (Table 1) showing significant inhibition values on both dermatophytes were EO04, EO46, and EO59 (from *Coridothymus capitatus*, *Thymus serpyllum,* and *Thymus vulgaris*, respectively), which at the concentration of 100 µg/mL showed growth inhibition values higher than 50%, in the case of EO46 well above 80% (92.31% on *M. canis* and 87.34% on *M. gypseum*).

Inspection of the three EOs revealed them to be constituted of 61%, 66%, and 44% of carvacrol (EO59) and thymol (EO04 and EO46), respectively. Two components were correctly reasonably highlighted from models ML1, ML3, and ML4. Moreover, the three EOs were all characterized by important percentages of p-cymene, γ-terpinene, and caryophyllene which were not indicated as strictly necessary for the *M. spp* inhibitory activity.

Interesting activity of EO09 and EO48 evidenced a selective inhibition greater than 50% against *M. gypseum*. Inspection for particular components to be correlated for *M. gypseum* activity did not reveal any particular component, except for caryophyllene which was the only one present in all five active EOs.

### 4.2. Machine Learning Classification Models

#### 4.2.1. ML Model on *M*. *canis*

As above described a RF-based ML model was developed on the 61 EOs associated to *M canis* bioactivity. According to the classification model, four compounds, namely limonene, thymol, linalool, and α-muurolene can be considered as those that mostly influence EO inhibition potency. In particular, limonene was calculated having the highest feature importance with a positive relevance with respect to the activity. Nevertheless, it could be evidenced that limonene is a compound which with a logP of 3.4 could show affinity to lipophilic environment such as dermatophyte membrane/wall, possibly acting/performing as a gate molecule, as previously reported for the modulation of bacteria biofilm production [1,2,3]. Assuming this as correct, it could be speculated that thymol (logP = 3.0) and linalool (logP = 3.0), slightly less hydrophobic than limonene and bearing a hydrophilic hydroxyl group, are the most important components, and maybe also others compounds, with the aid of limonene could contribute to the overall inhibitory activity by binding to specific cellular target(s). On the other hand, p-cymen-8-ol, cis-geraniol, β-pinene indicated as negatively influencing the EOs’ inhibitory activities could be ascribed to some adverse effects like the stimulation of *M. canis* growth. Inspection of EOs’ compositions endowed of some augmented *M. canis* growth effect (check the + and ++ signs on EO11, EO20, EO33, EO34, EO35, EO42, EO44 and EO53 samples in Table 1), actually all but EO33 and EO53 were found to contain, to some extents, β-pinene while only two cis-geraniol (EO11 and EO42) and just one contained p-cymen-8-ol (EO20).

From a survey on a list of published EOs assayed against *M. canis*, listed in the recent portal PyEO (eo.3d-qsar.com, 22 July 2021) the associated chemical composition was compared with those of the herein investigated 61 EOs. Nevertheless, the published EOs’ compositions were used to compile a test set to have a sort of predictive ability evaluation for model ML1. Interestingly, among the 23 published EOs (Supplementary Table S4) the percentage of chemical components on each test set EOs contained in the training set ranged from 0.91% to 18.35% thus showing a low chemical variation overlapping. Considering the low amount of mixture similarity with the training set, EOs model ML1 was applied to predict them as active or inactive. Although only three EOs out of 23 displayed an activity value lower than 100 μg/mL, the ML1 model was able to correctly predict as active, 2 of them among the active predicted TS1 EOs. As a consequence, a virtual success rate of more than 15% was obtained considering that 13 EOs of the test set were predicted as active. Considering the low composition similarities and in analogy with the pharmaceutical virtual screening campaign the success rate is of notable result [18,19,20], furthermore as a general application model ML1 was characterized by a predictive accuracy of 0.43 indicating that 43% of EO were correctly predicted as actives or inactives.

#### 4.2.2. ML Models on *M*. *gypseum*

Differently from *M canis* inhibitory activity-based ML1 model two linear models, ML3 and ML4 (Table 3) were the best describing the QCAR between the 61 EOs chemical compositions and the associated *M gypseum* growth-modulating potencies. Analysis of perturbation-based feature importance (Figure 4) was found to indicate that thymol, carvacrol, eugenol, pulegone, cis-geraniol, β-citral, α-citral, limonene, and eucalyptol as those mainly involved in modulating *M gypseum* growth. Inspection of the models’ linear coefficients (Figure 5) showed the high positive values attributed in descending order to thymol, carvacrol, eugenol, pulegone, cis-geraniol, p-cymene, trans-3-phenyl-2-propenal, β-citral, and α-citral, while negative coefficients were ascribed mainly to eucalyptol, limonene, and α-pinene. Taken together perturbation-based feature importance and linear coefficient clearly highlighted thymol and carvacrol to principally drive EOs’ *M gypseum* inhibition, while eucalyptol and limonene could be mainly responsible for EOs that induce dermatophyte growth (check + and ++ signs for EO02, EO03, EO07, EO13, EO16, EO23, EO27, EO28, EO38, EO40, EO41, EO44, EO55, EO56 samples in Table 1). A deeper inspection on EOs’ compositions actually revealed high percentages of eucalyptol in EO13, EO28, EO38, EO40, EO44, and EO55 while high content of limonene was listed in EO16 and EO41, being present in all *M. gypseum* growth-stimulating EOs in percentages ranging from about 0.27% to 95%. Again, here is highlighted the important role of limonene, but this time the data let to speculate that in sample with low-medium percentage its likely gate behavior is mainly associated to some negative compounds, whereas in high concentration limonene could exert a direct growth stimulation, being in EO27 and EO41 present at about 95%.

Tentative to build ML models with raw data were not successful in indicating DA as a tool to extract composition–activity relationships allowing an easier way to analyze the composition–activity relationships.

## 5. Conclusions

ML models were built on a dataset of 61 EOs biological activity on *M. canis* and *M. gypseum* showing excellent performances in terms of MCC (fitting, and CV). Results indicate DA as a powerful tool to avoid overfitting, improve model’s performance, and as for *M. gypseum,* to build models otherwise not available using the original raw dataset. In fact, in the case of *M. gypseum* the raw dataset did not allow to obtain ML models with acceptable classification ability. Moreover, feature importance has benefited from DA, leading to the identification of the most important compounds related to the anti-dermatophyte activity. Basing on the ML models on *M. canis* limonene, thymol, linalool, and α-muurolene are related to the increase of EOs inhibitory activity. While carvacrol, thymol, eugenol, pulegone, cis-geraniol, β-citral, α-citral, eucalyptol, and limonene are related to the inhibitory activity on *M. gypseum*. Finally, in an external prediction on already reported EOs biological activities on *M. canis* ML models were further validated reaching an accuracy of 43%, demonstrating once again the power of DA in this low data regime field. We believe these data very important to the field of drugs, foods, and cosmetics; indeed a first application of these results could be in the development of ingredients in the dermo-cosmetic field in view of the microbial species investigated and the impelling need of natural preserving and acting antimicrobial agents. 

## 6. Materials and Methods

### 6.1. Essential Oil and Chemical Composition Analysis

The 61 essential oil have been already chemically characterized by Papa et al. [3] and Di Martile et al. [6] therefore the reader is referred to their published articles.

### 6.2. Antimicrobial Assays

The two dermatophytes strains investigated in this study were purchased from the Centraal Bureau voor Schimmelcultures (CBS), *M. canis* (Iran) CBS 131110 strain and *M. gypseum* (Iran) CBS 130948 strain. All dermatophytes were maintained at 4 °C as agar slants on Sabouraud dextrose agar (SDA; Difco Laboratories, Inc., Detroit, MI, USA).

Antidermatophyte activity was determined as follows. Each test substance was dissolved in dimethylsulfoxide (DMSO) and aseptically mixed with sterile medium (SDA) at 45 °C to concentrations of 100 μg/mL.

The DMSO concentration in the final solution was adjusted to 0.1%. Controls were also prepared with equivalent concentrations (0.1% *v*/*v*) of DMSO. For experiments, cultures were obtained by transplanting mycelium disks (10 mm diameters) from a single mother culture in the stationary phase. They were incubated at 26 ± 1 °C on SDA on thin sheets of cellophane until the logarithmic growth phase.

Subsequently, the cultures were transferred to Petri plates with media containing 100 μg/mL of the single oil and incubated under growth conditions. The fungal growth was evaluated daily by measuring colony diameters (in millimeters) for seven days from the treatment onset.

The percent inhibition of growth was determined as the average of three different experiments.

### 6.3. Machine Learning Binary Classification

#### 6.3.1. Data Augmentation

EOs dataset was augmented by means of composition random perturbation while keeping the same bioactivity for each of augmented related EO. In particular for each EO all the components were randomly modified by adding or subtracting up to 15% to each essential oil components.

In the case of unbalanced augmentation, for each EO, 10 new “virtual” records were generated, while for the balanced process, being *w* the weight of the EO class, it was augmented w*10 times.

#### 6.3.2. Grid Search Model Optimization

All calculations were performed using the Python (version 3.9.5) programming language [21] by executing in-house code in the Jupyter Notebook platform [22]. All data were organized in a python Pandas dataframe [16] while all the machine learning algorithms and feature important analysis were done through python scripts using the scikit-learn library [16].

The classification models’ robustness validation was performed via five-fold cross-validation with 50 iterations.

To seek for the best model, a grid search was performed on the algorithms’ hyper-parameters for a total of 367 combinations (Table 4), three inhibition potency classification thresholds (15%, 20%, and 30%), and the use of raw dataset or two data augmented versions of it (balanced and unbalanced). Furthermore, while preprocessing, the application of PCA for features extraction was also tested using a total number of components with explained variance of 60% and 80%. This last experiment did not report significant results. Moreover, dimensionality reduction is incompatible with determining compound importance, which was one of the main purposes of this study.

By multiplying the combinations, the different search fields, a total of 4,050,000 models were generated.

## Figures and Tables

**Table 1:** Essential oil percentage of *M*. *canis* and *M*. *gypseum* growth inhibition at 100 μg/mL; +weak growth, ++ moderate growth.

EO ID	*M. Canis*	*M. Gypseum*	EO ID	*M. Canis*	*M. Gypseum*
**EO01**	7.20	24.39	**EO31**	8.28	6.03
**EO02**	5.60	+	**EO32**	27.22	42.24
**EO03**	2.40	+	**EO33**	+	10.34
**EO04**	67.13	85.45	**EO34**	+	0.00
**EO05**	4.20	4.55	**EO35**	+	3.70
**EO06**	4.90	15.45	**EO36**	7.98	24.07
**EO07**	6.67	+	**EO37**	11.56	46.62
**EO08**	4.00	3.00	**EO38**	0.58	+
**EO09**	26.00	72.00	**EO39**	33.53	33.11
**EO10**	11.45	11.21	**EO40**	0.00	+
**EO11**	+	0.00	**EO41**	1.14	+
**EO12**	1.53	13.08	**EO42**	+	0.00
**EO13**	4.07	++	**EO43**	3.42	3.82
**EO14**	12.79	20.16	**EO44**	+	+
**EO15**	2.91	4.65	**EO45**	0.85	3.05
**EO16**	2.60	+	**EO46**	92.31	87.34
**EO17**	5.84	2.06	**EO47**	0.00	11.39
**EO18**	1.30	2.06	**EO48**	44.62	51.27
**EO19**	6.96	17.14	**EO49**	8.63	5.00
**EO20**	+	3.81	**EO50**	8.63	4.38
**EO21**	3.16	11.43	**EO51**	2.88	5.63
**EO22**	24.85	26.67	**EO52**	27.45	41.25
**EO23**	2.96	+	**EO53**	+	9.38
**EO24**	11.24	12.38	**EO54**	14.38	40.63
**EO25**	5.75	2.04	**EO55**	8.64	+
**EO26**	12.64	25.51	**EO56**	2.47	+
**EO27**	1.15	+	**EO57**	8.64	13.53
**EO28**	1.78	+	**EO58**	16.28	30.68
**EO29**	6.51	3.70	**EO59**	53.49	69.89
**EO30**	1.78	5.56	**EO60**	20.93	44.32
			**EO61**	23.84	40.91

**Table 2:** Classification model results for *M*. *canis* with and without data augmentation.

Dataset	Model ^1^	Experiments ^2^	Accuracy ^3^	MCC ^4^	F_1_ Score ^5^	Accuracy_CV_^6^	MCC_CV_^7^	F_1_ Score_CV_^8^
DA Dataset ^9^	ML1	1050	1	1	1	0.95	0.84	0.97
Raw Dataset ^10^	ML2	61	1	1	1	0.93	0.77	0.96

^1^ model ID. ^2^ number of experiments used for training and cross-validation. ^3^ Accuracy is a function that computes the fraction of correct recalculated/predicted classes. ^4^ Matthew correlation coefficient. ^5^ F1 Score is the harmonic mean of the precision and recall. ^6^ The cross-validated Accuracy. ^7^ The cross-validated MCC. ^8^ The cross-validated F1 Accuracy. ^9^ The augmented and balanced dataset. ^10^ The original dataset.

**Table 3:** Classification model results for *M. canis* with data augmentation.

ML Algorithm	Model ^1^	Experiments ^2^	Accuracy ^3^	MCC ^4^	F_1_ Score ^5^	Accuracy_CV_^6^	MCC_CV_^7^	F_1_ Score_CV_^8^
LR	ML3	1050	0.99	0.99	0.99	0.80	0.93	0.96
SVM	ML4	1050	0.95	0.98	0.99	0.80	0.93	0.96

^1^ model ID. ^2^ number of experiments used for training and cross-validation. ^3^ Accuracy is a function that computes the fraction of correct recalculated/predicted classes. ^4^ Matthew correlation coefficient. ^5^ F1 Score is the harmonic mean of the precision and recall. ^6^ The cross-validated Accuracy. ^7^ The cross-validated MCC. ^8^ The cross-validated F1 Accuracy.

**Table 4:** Hyper-parameter ranges for the grid search. Summing all the combinations, a total of 367 different models is obtained.

ML Algorithm	Hyperparameters	Ranges	Number of Combinations
LR	C ^1^	(0.001, 0.01, 1)	3
KNN	n_neighbors	(1, 2, 3, 4, 5)	5
RF	n_estimators	(1, 2, 3, 4, 5, 6, 7, 8, 9, 10)	10 × 10 × 2 = 200
	max_depth ^3^	(2, 5, 6, 7, 8, 9, 10, None)	
	class_weight ^4^	(‘balanced’, None)	
GB	n_estimators	(1, 2, 3, 4, 5, 6, 7, 8, 9, 10)	10 × 3 × 5 = 150
	learning_rate	(0.1, 0.01, 0.05)	
	max_features ^2^	(‘auto’, ‘sqrt’, ‘log2’, 10, 15)	
SVM	C ^1^	(0.001, 0.01, 1)	3 × 3 = 9
	kernel	(‘linear’, ‘rbf’,‘poly’)	

^1^ Regularization coefficient. ^2^ Maximum number of features used by the algorithm. ‘sqrt’ and ‘log2’ mean calculating the square root and logarithm in basis 2 of the total number of features, respectively. ^3^ Maximum depth of the generated trees. ^4^ Whether the model has to train on balanced class weights or unbalanced ones.

## Supplementary Materials

The following are available online. Figure S1: Full RF first decision tree, Figure S2: Full RF sec-ond decision tree; Table S1: Essential oils IDs and associated plants. As the essential oil were of commercial source part plants from which the distillation was done were not always indicated; Table S2: Optimized hyperparameters for the ML algorithms applied to the M. canis dataset; Ta-ble S3: Optimized hyperparameters for the ML algorithms applied to the M. gypseum dataset; Table S4: Composition of the test set for model ML1.

## Sample Availability

Samples of the compounds are available from the authors.
