# Supplementary material for: Machine Learning Data Augmentation as a Tool to Enhance Quantitative Composition–Activity Relationships of Complex Mixtures. A New Application to Dissect the Role of Main Chemical Components in Bioactive Essential Oils
Source: Molecules. 2021 Oct 17;26(20):6279. doi: 10.3390/molecules26206279 (PMC8537614; doi:10.3390/molecules26206279)
Supplement: Supplementary file 1 [file molecules-26-06279-s001.zip › Tables_S1_and_S2.pdf]

Table S1. Optimized hyperparameters for the ML algorithms applied to the *M. canis* dataset.

**model_name**	RF	SVC	GB	LR	KNN
**preprocess_fn**	baug10	baug10	baug10	baug10	baug10
**model**	bootstrap=True<br>ccp_alpha=0.0<br>class_weight=None<br>criterion='gini'<br>max_depth=None<br>max_features='auto'<br>max_leaf_nodes=None<br>max_samples=None<br>min_impurity_decrease=0.0<br>min_impurity_split=None<br>min_samples_leaf=1<br>min_samples_split=2<br>min_weight_fraction_leaf=0.0<br>n_estimators=2<br>n_jobs=None<br>oob_score=False<br>random_state=None<br>verbose=0<br>warm_start=False	C=0.1<br>break_ties=False<br>cache_size=200<br>class_weight=None<br>coef0=0.0<br>decision_function_shape='ovr'<br>degree=3<br>gamma='scale'<br>kernel='linear'<br>max_iter=-1<br>probability=False<br>random_state=None<br>shrinking=True<br>tol=0.001<br>verbose=False	ccp_alpha=0.0<br>criterion='friedman_mse'<br>init=None<br>learning_rate=0.1<br>loss='deviance'<br>max_depth=3<br>max_features='log2'<br>max_leaf_nodes=None<br>min_impurity_decrease=0.0<br>min_impurity_split=None<br>min_samples_leaf=1<br>min_samples_split=2<br>min_weight_fraction_leaf=0.0<br>n_estimators=7<br>n_iter_no_change=None<br>presort='deprecated'<br>random_state=None<br>subsample=1.0<br>tol=0.0001<br>validation_fraction=0.1<br>verbose=0<br>warm_start=False	C=0.1<br>class_weight=None<br>dual=False<br>fit_intercept=True<br>intercept_scaling=1<br>l1_ratio=None<br>max_iter=100<br>multi_class='auto'<br>n_jobs=None<br>penalty='l2'<br>random_state=None<br>solver='lbfgs'<br>tol=0.0001<br>verbose=0<br>warm_start=False	algorithm='auto'<br>leaf_size=30<br>metric='minkowski'<br>metric_params=None<br>n_jobs=None<br>n_neighbors=1<br>p=2<br>weights='uniform'
**threshold**	20	20	20	20	20
**component_min**	0	0	0	0	0
**kfold**	5	5	5	5	5
**pc**	0	0	0	0	0
**mcc_cv**	0.84	0.77	0.77	0.774	0.66
**acc_cv**	0.95	0.93	0.93	0.937	0.88
**mcc_fit**	1	0.95	0.95	0.952	1
**acc_fit**	1	0.98	0.98	0.989	1
**pca**					
**idx**	36531	36339	36434	36699	36692

Table S2. Optimized hyperparameters for the ML algorithms applied to the *M. gypseum* dataset.

**model_name**	SVC	LR	GB	RF	KNN
**preprocess_fn**	baug10	baug10	baug10	baug10	baug10
**model**	C=0.1<br>break_ties=False<br>cache_size=200<br>class_weight=None<br>coef0=0.0<br>decision_function_shape='ovr'<br>degree=3<br>gamma='scale'<br>kernel='linear'<br>max_iter=-1<br>probability=False<br>random_state=None<br>shrinking=True<br>tol=0.001<br>verbose=False	C=0.1<br>class_weight=None<br>dual=False<br>fit_intercept=True<br>intercept_scaling=1<br>l1_ratio=None<br>max_iter=100<br>multi_class='auto'<br>n_jobs=None<br>penalty='l2'<br>random_state=None<br>solver='lbfgs'<br>tol=0.0001<br>verbose=0<br>warm_start=False	ccp_alpha=0.0<br>criterion='friedman_mse'<br>init=None<br>learning_rate=0.01<br>loss='deviance'<br>max_depth=3<br>max_features=10<br>max_leaf_nodes=None<br>min_impurity_decrease=0.0<br>min_impurity_split=None<br>min_samples_leaf=1<br>min_samples_split=2<br>min_weight_fraction_leaf=0.0<br>n_estimators=10<br>n_iter_no_change=None<br>presort='deprecated'<br>random_state=None<br>subsample=1.0<br>tol=0.0001<br>validation_fraction=0.1<br>verbose=0<br>warm_start=False	(bootstrap=True<br>ccp_alpha=0.0<br>class_weight='balanced'<br>criterion='gini'<br>max_depth=9<br>max_features='auto'<br>max_leaf_nodes=None<br>max_samples=None<br>min_impurity_decrease=0.0<br>min_impurity_split=None<br>min_samples_leaf=1<br>min_samples_split=2<br>min_weight_fraction_leaf=0.0<br>n_estimators=5<br>n_jobs=None<br>oob_score=False<br>random_state=None<br>verbose=0<br>warm_start=False	algorithm='auto'<br>leaf_size=30<br>metric='minkowski'<br>metric_params=None<br>n_jobs=None<br>n_neighbors=1<br>p=2<br>weights='uniform'
**threshold**	30	30	30	30	30
**component_min**	0	0	0	0	0
**kfold**	5	5	5	5	5
**pc**	0	0	0	0	0
**mcc_cv**	0.80	0.80	0.75	0.69	0.61
**acc_cv**	0.94	0.93	0.92	0.90	0.86
**mcc_fit**	0.95	1	0.45	1	1
**acc_fit**	0.98	1	0.64	1	1
**pca**					
**idx**	36339	36699	36485	36586	36692
