# Supplementary material for: Machine Learning Data Augmentation as a Tool to Enhance Quantitative Composition–Activity Relationships of Complex Mixtures. A New Application to Dissect the Role of Main Chemical Components in Bioactive Essential Oils
Source: Molecules. 2021 Oct 17;26(20):6279. doi: 10.3390/molecules26206279 (PMC8537614; doi:10.3390/molecules26206279)
Supplement: Supplementary file 1 [file molecules-26-06279-s001.zip › Table_S3.pdf]

Table S3. Composition of the test set for model ML1

Extract[1]	doi	Activity Type[2]	Potency	Unit	# Components[3]	Experimental Class[4]	Predicted Class	Consensus[5]
260	10.1016/j.jep.2015.10.021	MIC	0.125	mg/ml	3	I	A	0
265	10.1016/j.fct.2014.11.009	MBC	0.64	mg/ml	17	I	A	0
266	10.1016/j.fct.2014.11.009	MBC	128	mg/ml	21	I	A	0
481	10.1016/j.indcrop.2013.08.033	MIC	0.08	ul/ml	36	A	A	1
697	10.3390/molecules22101595	MBC	0.64	ul/ml	8	I	I	1
758	10.1080/14786419.2017.1292266	MIC	0.64	ul/ml	21	I	A	0
794	10.3390/molecules23020396	MIC	16.26	mg/ml	30	I	I	1
795	10.3390/molecules23020396	MIC	17.04	mg/ml	31	I	A	0
941	10.1080/0972060X.2017.1341344	MIC	0.3	ul/ml	17	I	A	0
1289	10.3389/fphar.2019.00446	MIC	0.57	mg/ml	38	I	I	1
1290	10.3389/fphar.2019.00446	MIC	0.57	mg/ml	33	I	A	0
1510	10.1016/j.jep.2016.06.023	MIC	0.64	ul/ml	18	I	I	1
1544	10.1016/j.jep.2016.10.076	MIC	0.945	ul/ml	15	I	A	0
1577	10.1016/j.indcrop.2014.08.004	MIC	0.16	ul/ml	37	I	I	1
1666	10.1080/14786419.2014.939085	MIC	0.48	ul/ml	26	I	A	0
1738	10.1016/j.jep.2012.05.032	MIC	1000	ug/ml	13	I	I	1
1760	10.1080/10412905.2004.9698654	MIC	6	ul/ml	40	I	A	0
1761	10.1080/13880200490511936	MFC	12.5	ul/ml	13	I	A	0
1854	10.1016/j.indcrop.2018.01.024	MIC	0.32	ul/ml	12	I	I	1
1855	10.1016/j.indcrop.2018.01.024	MIC	0.64	ul/ml	27	I	I	1
1995	10.1080/0972060X.2007.10643588	MIC	0.0625	mg/ml	2	A	A	1
2083	10.1080/10412905.2015.1006737	MIC	1000	ug/ml	16	I	I	1
2157	10.1155/2020/5679408	EC50	0.06	ul/ml	17	A	I	0

[1] Extract ID as found in eo.3d-qsar.com
[2] The activity found in the corresponding article doi
[3] Number of components overlapping those of the *M canis* training set
[4] I = Inactive, A = Active
[5] 0 uncorrectly predicted, 1 = correctly predicted
